# *ALDH1A1* mRNA expression in association with prognosis of triple-negative breast cancer

**DOI:** 10.18632/oncotarget.6023

**Published:** 2015-10-08

**Authors:** Yan Liu, Michelle Baglia, Ying Zheng, William Blot, Ping-Ping Bao, Hui Cai, Sarah Nechuta, Wei Zheng, Qiuyin Cai, Xiao Ou Shu

**Affiliations:** ^1^ Division of Epidemiology, Department of Medicine, Vanderbilt Epidemiology Center, Vanderbilt University Medical Center, Nashville, TN, USA; ^2^ Department of Cancer Prevention and Control, Shanghai Municipal Center for Disease Control and Prevention, Shanghai, China; ^3^ International Epidemiology Institute, Rockville, MD, USA

**Keywords:** ALDH1, triple-negative breast cancer, mRNA expression, prognosis

## Abstract

*ALDH1* is a crucial element in the retinoic acid signaling pathway regulating the self-renewal and differentiation of normal stem cells, and may play an important role in cancer progression. However, research on *ALDH1* gene expressionand breast cancer prognosis has yielded conflicting results. We evaluated the association between tumor tissue *ALDH1A1*/*ALDH1A3* mRNA expression and triple-negative breast cancer (TNBC) prognosis in the Shanghai Breast Cancer Survival Study (SBCSS, N=463), Nashville Breast Health Study (NBHS, N=86), and Southern Community Cohort Study (SCCS, N=47). Gene expression was measured in RNA isolated from breast cancer tissues. In the SBCSS, higher *ALDH1A1* mRNA level was associated with improved disease-free (HR=0.87, 95% CI: 0.80-0.95, per log unit change) and overall survival (HR=0.85, 95% CI: 0.78-0.93 per log unit change) independent of age at diagnosis, TNM stage and treatment. We replicated the findings for overall survival in the NBHS and SCCS (HR = 0.27, 95% CI: 0.10-0.73) and for disease-free survival by a meta-analysis of four publicly-available gene expression datasets (HR = 0.86, 95% CI: 0.76-0.97). No significant association was found for *ALDH1A3*. Our study suggests high expression of *ALDH1A1* mRNA in tumor tissues may be an independent predictor of a favorable TNBC outcome.

## INTRODUCTION

Aldehydes, which accumulate during the metabolism and biotransformation of chemicals and drugs, are reactive electrophilic compounds which are harmful to the organism [1]. Aldehyde dehydrogenases (ALDH) are a family of enzymes that catalyze aldehyde conversion into carboxylic acids via NAD(P)^+^-dependent oxidation [2]. In addition to detoxifying aldehydes, ALDH enzymes have multiple other functions, such as nitrate reductase activity [1]. These enzymes are present in various human tissues, with the highest concentration in the liver, and are also found in stem cells [3]. High ALDH activity has been detected in hematopoietic stem/progenitor cells [4, 5], and inhibition of ALDH activity has been shown to impair the differentiation of hematopoietic stem cells [6]. Within cells, ALDH is found in cytosols, nuclei, mitochondria, and endoplasmic reticulum. Nineteen ALDH family members have been identified in humans, including *ALDH1A1, ALDH1A3, ALDH2, ALDH3A1,* and *ALDH4A*1 [1].

*ALDH1A1* has been suggested as a breast cancer stem cell marker [7, 8]. However, contradictory findings on the role of *ALDH1A1* in predicting the prognosis of breast cancer patients have been reported. Some studies have shown that *ALDH1A1* protein expression is associated with late-stage cancer, large tumor size, chemoresistance, and poor prognosis [7, 9, 10], while other studies have found that *ALDH1A1* protein levels do not predict breast cancer survival [11, 12]. In addition, one study reported that high levels of *ALDH1A1* in tumor stromal tissues are associated with better clinical outcomes [13]. Considered together, these study findings are inconclusive in determining whether the expression of *ALDH1A1*, either as mRNA or as a protein, can predict clinical outcomes in breast cancer patients. In addition, no studies have specifically evaluated the role of *ALDH1A1* in predicting prognosis of triple-negative breast cancer (TNBC: estrogen receptor negative (ER-), progesterone receptor negative (PR-), human epidermal growth factor receptor 2 negative (HER2-)).

In this study, we analyzed the association between mRNA expression of the *ALDH1A1* gene in tumor tissues, and the clinical outcomes in patients with TNBC in three cohorts of breast cancer patients. We also evaluated the association between *ALDH1A3* gene expression and TNBC prognosis because one study had suggested that *ALDH1A3* expression can predict metastasis in breast cancer patients [11]. In addition, we validated our findings using 4 publicly-available gene expression data sets.

## RESULTS

**Figure 1:**
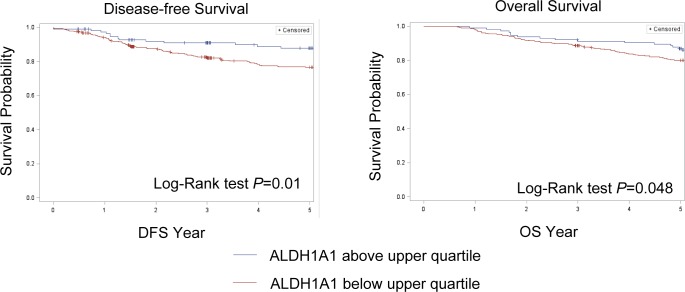
Kaplan-Meier curves Participants with *ALDH1A1* mRNA expression levels below the upper quartile have a significantly worse prognosis compared to participants with *ALDH1A1* mRNA expression levels in the upper quartile.

**Figure 2:**
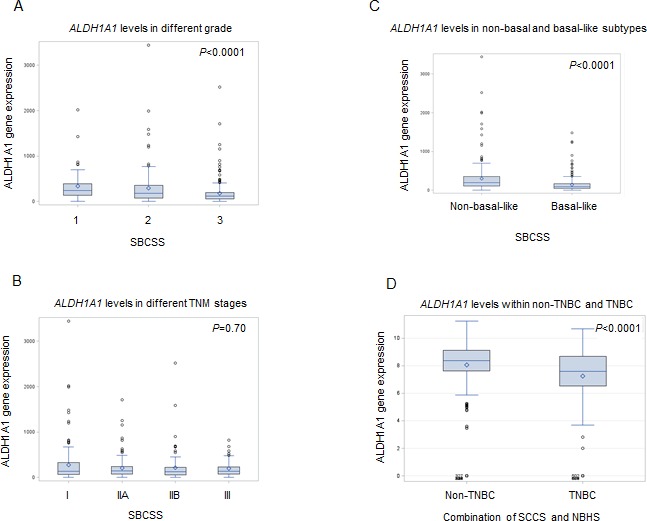
Correlation of *ALDH1A1* gene expression with tumor grade A., stage B., basal-like breast cancer subtype C. and TNBC D

In the SBCSS, TNBC patients with expression levels of the *ALDH1A1* gene above the median had better disease-free survival (DFS) (*P* = 0.01) and overall survival (OS) (*P* = 0.048) than those with expression levels of *ALDH1A1* below the median (Figure 1). In the multivariate analysis, adjusted for age at diagnosis and TNM stage, one log unit increment of the *ALDH1A1* gene expression was associated with DFS (HR = 0.87, 95% CI: 0.80-0.95) and OS (HR = 0.85, 95% CI: 0.78-0.93) (Table 2). The association remained largely unchanged after further adjustment for radiotherapy treatment, chemotherapy treatment, and basal-like breast cancer subtype (Table 2). Analyses by quartile or median cut points of expression levels of the *ALDH1A1* gene revealed a similar pattern: higher *ALDH1A1* expression was associated with better DFS and OS, although not all associations were statistically significant, particularly when subtypes of TNBC were adjusted for. No association of *ALDH1A3* gene expression levels with DFS and OS was observed in the SBCSS (Table 2). In addition, we found that patients with higher grade tumors had a lower level of *ALDH1A1* mRNA expression (Figure 2A); no association was found for TNM stages (Figure 2B). SBCSS participants with basal-like TNBC had lower *ALDH1A1* expression levels than participants with non-basal-like TNBC (Figure 2C).

**Table 1:** Characteristics of participants in the SBCSS, NBHS, and SCCS cohorts

Study	Characteristics	Number	5-yr DFS/OS for NBHS and SCCS	*P-value*
SBCSS	No. of cases	463	79.3%	
	Age at diagnosis, median (range)	51.6 (26.1-74.3)		
	TNM stage			<0.001
	I	143	88.4%	
	IIA	165	84.2%	
	IIB	93	70.0%	
	III	46	51.9%	
	Unknown	16	80.4%	
	Grade			0.34
	1	54	87.7%	
	2	148	79.3%	
	3	259	77.8%	
	Unknown	2	50.0%	
	Basal-like			0.03
	Yes	217	75.4%	
	No	246	82.8%	
NBHS	No. of cases	86	89.2%	
	Age at diagnosis, median (range)	52.0 (28.0-75.0)		
	TNM stage			0.31
	I	38	97.3%	
	IIA	6	80.0%	
	IIB	22	81.8%	
	III	5	100.0%	
	Unknown	15	78.6%	
	Grade			0.65
	1-2	12	91.7%	
	3	68	89.3%	
	Unknown	6	83.3%	
	Basal-like			0.12
	Yes	72	87.0%	
	No	14	100.0%	
	No. of deaths	11		
SCCS	No. of cases	47	82.5%	
	Age at diagnosis, median (range)	56.0 (44.0-74.0)		
	TNM stage			<.0001
	I	19	94.7%	
	IIA	6	100.0%	
	IIB	12	83.3%	
	III	5	40.0%	
	Unknown	5	60.0%	
	Grade			0.41
	1-2	8	87.5%	
	3	29	78.4%	
	Unknown	10	90.0%	
	Basal-like			0.80
	Yes	42	80.4%	
	No	5	100.0%	

**Table 2:** Association of *ALDH1A1/ALDH1A3* with disease-free survival and overall survival in the SBCSS

	Disease-free survival			Overall survival		
	HR^1^ (95% CI)	HR^2^ (95% CI)	HR^3^ (95% CI)	HR^1^ (95% CI)	HR^2^ (95% CI)	HR^3^ (95% CI)
***ALDH1A1***						
Continuous	0.87 (0.80-0.95)	0.87 (0.80-0.95)	0.88 (0.80-0.96)	0.85 (0.78-0.93)	0.85 (0.79-0.93)	0.86 (0.79-0.94)
*P*	0.002	0.002	0.006	0.0002	0.0002	0.001
Q1 (lower quartile)	1.00 (reference)	1.00 (reference)	1.00 (reference)	1.00 (reference)	1.00 (reference)	1.00 (reference)
Q2	0.71 (0.41-1.23)	0.72 (0.41-1.24)	0.76 (0.43-1.32)	0.51 (0.29-0.89)	0.52 (0.30-0.91)	0.57 (0.33-1.00)
Q3	0.65 (0.38-1.11)	0.67 (0.39-1.16)	0.75 (0.42-1.32)	0.64 (0.38-1.06)	0.66 (0.40-1.10)	0.76 (0.44-1.30)
Q4	0.38 (0.20-0.73)	0.40 (0.20-0.77)	0.44 (0.22-0.88)	0.41 (0.23-0.74)	0.44 (0.24-0.79)	0.51 (0.27-0.94)
*P*_trend_	0.004	0.007	0.03	0.007	0.01	0.07
Below median	1.00 (reference)	1.00 (reference)	1.00 (reference)	1.00 (reference)	1.00 (reference)	1.00 (reference)
Above median	0.61 (0.39-0.94)	0.63 (0.41-0.97)	0.69 (0.44-1.09)	0.71 (0.47-1.07)	0.73 (0.49-1.10)	0.83 (0.54-1.27)
*P*	0.02	0.04	0.11	0.10	0.13	0.39
***ALDH1A3***						
Continuous	1.00 (0.91-1.09)	0.99 (0.90-1.08)	0.97 (0.89-1.07)	0.98 (0.90-1.06)	0.97 (0.89-1.06)	0.96 (0.88-1.04)
*P*	0.94	0.80	0.61	0.60	0.51	0.31
Q1 (lower quartile)	1.00 (reference)	1.00 (reference)	1.00 (reference)	1.00 (reference)	1.00 (reference)	1.00 (reference)
Q2	0.47 (0.24-0.90)	0.41 (0.21-0.80)	0.39 (0.20-0.76)	0.57 (0.30-1.06)	0.50 (0.27-0.94)	0.48 (0.25-0.90)
Q3	0.90 (0.52-1.57)	0.83 (0.48-1.45)	0.77 (0.44-1.35)	1.10 (0.65-1.86)	1.033 (0.61-1.75)	0.95 (0.56-1.62)
Q4	1.01 (0.58-1.74)	1.03 (0.60-1.77)	0.99 (0.57-1.70)	0.95 (0.55-1.64)	0.96 (0.56-1.66)	0.92 (0.54-1.59)
*P*_trend_	0.53	0.45	0.54	0.61	0.51	0.62
Below median	1.00 (reference)	1.00 (reference)	1.00 (reference)	1.00 (reference)	1.00 (reference)	1.00 (reference)
Above median	1.33 (0.88-2.02)	1.37 (0.91-2.08)	1.34 (0.88-2.03)	1.32 (0.89-1.98)	1.38 (0.92-2.06)	1.33 (0.89-1.99)
*P*	0.18	0.14	0.17	0.17	0.12	0.17

1 Adjusted for age at diagnosis and TNM stage

2 Adjusted for age at diagnosis, TNM stage, and radiotherapy and chemotherapy treatment

3 Adjusted for age at diagnosis, TNM stage, radiotherapy and chemotherapy treatment, and basal-like subtype

A similar association pattern was observed in the SCCS and NBHS, although not all point estimates were statistically significant, likely due to the small sample sizes of the individual studies (Table 3). When the two studies were combined, *ALDH1A1* gene expression level was positively, but not statistically significantly, associated with OS (HR = 0.88, 95% CI: 0.72-1.09). When categorized into two groups, participants with *ALDH1A1* expression levels above the median had a reduced risk of OS (HR = 0.27, 95% CI: 0.10-0.73). Similarly, no association of *ALDH1A3* gene expression level with OS was observed in the SCCS and NBHS (Table 3). In an analysis of overall survival based on the combined data from all three cohorts, we found that *ALDH1A1* gene expression level was statistically significantly associated with OS regardless of whether it was treated as a continuous variable (HR = 0.86, 95% CI: 0.79-0.93) or categorized by median cuts (HR = 0.64, 95% CI: 0.44-0.93). Similarly, we found no association between *ALDH1A3* gene expression level and OS in the combined analysis (Table 3). Information on DFS was not available for SCCS and NBHS participants.

**Table 3:** Association of *ALDH1A1/ALDH1A3* with overall survival in the SCCS and NBHS

Overall survival				
	SCCS (n=47)	NBHS (n=86)	Pooled (SCCS and NBHS n=133)	Pooled (SCCS, NBHS, and SBCSS n=596)
	HR^1^ (95% CI)	HR^1^ (95% CI)	HR^2^ (95% CI)	HR^2^ (95% CI)
***ALDH1A1***				
Continuous	0.54 (0.30-0.97)	0.96 (0.75-1.24)	0.88 (0.72-1.09)	0.86 (0.79-0.93)
*P*	0.04	0.77	0.23	0.0001
Below median	1.00 (reference)	1.00 (reference)	1.00 (reference)	1.00 (reference)
Above median	0.058 (0.005-0.70)	0.42 (0.11-1.57)	0.27 (0.10-0.73)	0.64 (0.44-0.93)
*P*	0.02	0.20	0.01	0.02
***ALDH1A3***				
Continuous	0.68 (0.33-1.37)	1.21 (0.83-1.76)	1.08 (0.84-1.39)	1.00 (0.92-1.08)
*P*	0.28	0.32	0.56	0.89
Below median	1.00 (reference)	1.00 (reference)	1.00 (reference)	1.00 (reference)
Above median	0.85 (0.12-5.98)	1.23 (0.36-4.27)	1.11 (0.45-2.76)	1.21 (0.83-1.75)
*P*	0.87	0.74	0.82	0.32

1 Adjusted for age at diagnosis and TNM stage

2 Adjusted for age at diagnosis, TNM stage, and study

Meta-analyses on the association between *ALDH1A1/ALDH1A3* gene expression and DFS in 4 publicly-available TNBC datasets with 347 samples were conducted based on continuous variable and median cut points. Again, we found that expression level of the *ALDH1A1* gene was positively associated with DFS (HR = 0.86, 95% CI: 0.76-0.97, based on analysis of continuous scale; and HR = 0.58, 95% CI: 0.39-0.85, based on median cut point). *ALDH1A3* gene expression level was not associated with DFS (Table 4).

**Table 4:** Meta-analysis of the association of *ALDH1A1/ALDH1A3* with disease-free survival in four publicly available TNBC datasets

	Disease-Free Survival	
	HR^1^ (95% CI)	*P*
***ALDH1A1***		
Continuous	0.86 (0.76 - 0.97)	0.014
Below median	1.00 (reference)	
Above median	0.58 (0.39 - 0.85)	0.005
***ALDH1A3***		
Continuous	0.98 (0.87 - 1.10)	0.71
Below median	1.00 (reference)	
Above median	0.94 (0.65 - 1.37)	0.75

Datasets included: GSE25065 (n=64); GSE25055 (n=95); GSE21653 (n=87); GSE10886 (n=101)

1 Adjusted for age at diagnosis and TNM stage

HR and P were derived from fixed effect model

Finally, in the combined data from the SCCS and NBHS studies, we found that TNBC patients had lower expression levels of the *ALDH1A1* gene than did non-TNBC patients (Figure 2D).

## DISCUSSION

TNBC is an aggressive breast cancer subtype with limited treatment options. Identification of new biomarkers for prognosis is urgently needed. It has been suggested that ALDH1 is a biomarker for normal and malignant mammary stem cells [7]. Human mammary epithelial cells with high ALDH activity have stem cell characteristics and have the potential to form tumors *in vivo*. In a study of 577 cancer tissues of all types of breast cancer combined, ALDH1, detected by immunohistochemical (IHC) staining, was correlated with poorer survival [7]. In our study, we found that *ALDH1A1* expression was higher for receptor-positive, low-grade, and non-basal like TNBC tumor tissue. Not taking those clinical predictors into consideration in analysis could result in a false inverse association between *ALDH1A1* and cancer prognosis.

*ALDH1A1* can inactivate integral agents of chemotherapy; therefore, it has been postulated that breast cancer patients with high *ALDH1A1* expression may have an increased risk of recurrence [14]. Two other studies on all types of breast cancer combined also reported that the *ALDH1A1* protein was a potential predictive marker of early local tumor recurrence and distant metastasis [9, 10]. However, different results were reported by other studies. These include one study reporting that *ALDH1A3,* rather than *ALDH1A1*, contributes to the ALDH activity of cancer stem cells in tissues and cell lines [11], and another study observing no correlation between ALDH and breast cancer stem cells [15]. High expression of ALDH1 in stromal tissues was found to be associated with better DFS and OS in another study [13]. Key limitations of previous studies include not adjusting for confounding factors, such as ER/PR status or TNM stage, and not taking into consideration the positive staining in tumor cells and stromal tissues.

In our study, we sought to evaluate the association between *ALDH1A1/ALDH1A3* mRNA expression levels and TNBC outcomes using three population-based cohorts, following an identical lab protocol for each. Total RNA was extracted from breast cancer tissues with at least 80% of the tissues from tumor cells, to reduce the effects of stromal cells on the analyses. We adjusted for age at diagnosis, TNM stage, chemotherapy and radiotherapy treatments, and basal-like breast cancer subtype. Results from these three independent studies demonstrated that high *ALDH1A1* gene expression level is associated with reduced breast cancer recurrence and total mortality in patients with TNBC, independent of age at diagnosis and TNM stage. In the SBCSS, additional adjustment for common treatment types and breast cancer basal-like subtypes did not materially alter the observed associations. However, the association for the dichotomized *ALDH1* level (by median cut) lost its significance when adjustment for TNBC subtype was made, probably due to reduced statistical power from collapsing the top two quartiles that are associated with different effect sizes. Furthermore, meta-analysis using 4 publicly-available TNBC datasets validated the association between high *ALDH1A1* gene expression level and improved DFS. Our results suggest that *ALDH1A1* mRNA expression in tumor tissue may be an independent predictor of TNBC recurrence and mortality.

Although we have attempted to include tissue with at least 80% of tumor cells in our study, we cannot completely remove the stromal cells. If cancer stem cells, which presumably have a high mRNA expression level of *ALDH1A1* and account for 3-4% of breast cancer cells, are the main source of elevated ALDH1A1 in tumor tissue [7], then the vast majority of *ALDH1A1* mRNA measured in our study could still come from stromal cells. Simultaneous measurement of mRNA and protein levels in the same tissue sample would help answer this question and should be considered in the future studies. Other limitations of our study include lack of information on recurrence and the relatively small sample sizes from the NBHS and SCCS. The strengths of our study are its inclusion of multiple independent cohorts and its collection of a large number of TNBC cases. In addition, we adjusted for a wide array of potential confounding factors, including age at diagnosis, TNM stage, chemotherapy and radiotherapy treatments, and basal-like breast cancer subtype.

In summary, our data indicate that tumor tissue *ALDH1A1* mRNA expression level may be an independent biomarker of prognosis in TNBC patients.

## MATERIALS AND METHODS

### Study population

Participants in this study were drawn from three studies: the Shanghai Breast Cancer Survival Study (SBCSS, *n* = 463), the Nashville Breast Health Study (NBHS, *n* = 86), and the Southern Community Cohort Study (SCCS, *n* = 47) (Table 1). Only patients with TNM stage I-III TNBC were included in the present study. A description of the participants has been published elsewhere [16-19].

Briefly, the SBCSS is a population-based cohort study of 5,042 incident breast cancer survivors, aged 20 to 75 years, recruited to the study approximately 6 months following cancer diagnosis [16]. In-person interviews and record linkages were conducted to collect information on demographics, lifestyle factors, clinical characteristics, and disease outcome (recurrence and morality). The demographic and clinical predictors for breast cancer among these participants with TNBC were previously reported [17]. Medical charts from each patient's initial diagnostic hospital were reviewed to gather information on tumor characteristics (including stage and grade), first-line treatments, and ER/PR status. HER2 status was assessed in the Vanderbilt Molecular Epidemiology Laboratory [17, 20]. Tumor sections were collected from the diagnostic hospitals, resulting in tumor tissue samples from 463 participants being included in the current study.

The NBHS is a population-based, case-control study of incident breast cancer among 2,726 women, aged 25 to 75 years, who were newly diagnosed with primary breast cancer between 2001 and 2011 [18, 21]. Information on demographic, anthropometric, medical, reproductive, and other characteristics was ascertained through telephone interview by trained interviewers using a structured questionnaire. Breast cancer diagnosis information was derived from medical and pathology records, including types and results of diagnostic tests, histopathology, tumor stage, tumor grade, and hormone receptor status. Mortality information was obtained by linkage to the National Death Index through December 31^st^, 2011. Eighty-six TNBC cases with tissue samples were included in the current study.

The SCCS is a population-based, prospective cohort study of 85,806 participants, aged 40 to 79 years, who were recruited between 2002 and 2009 from 12 southeastern states in the US [19, 22]. Ascertainment of incident breast cancer cases among SCCS participants was obtained through annual linkage of the cohort with the 12 state cancer registries that cover the SCCS catchment area. Mortality information was obtained through linkage with the National Death Index. Information on ER, PR, and HER2 status, as well as first-line treatment, was obtained from these tumor registries. Forty-seven TNBC cases with tissue samples were included in the current study.

### Gene expression analysis

Participants' hematoxylin and eosin (H&E) slides were reviewed by a study pathologist. Tumor tissue was dissected to ensure that samples contained more than 80% tumor cells for RNA extraction [17]. Total RNA was isolated and purified using miRNeasy FFPE Kit (Qiagen, Valencia, CA), and quality and quantity were checked with Nanodrop and an Agilent BioAnalyzer. Expression levels of *ALDH1A1* and *ALDH1A3* genes were measured as part of a large gene expression effort. A custom-designed nCounter Gene Expression CodeSet profiling of 311 selected gene targets using NanoString nCounter technology was performed following the NanoString standard protocol. Quality control and normalization of gene expression data protocol has been described in detail elsewhere [17]. Briefly, the R package NanoStringNorm (version 1.1.16) was used for quality control and expression normalization with five housekeeping genes (*ACTB*, *RPLP0*, *MRPL19*, *SF3A1* and *PSMC4*). The expression data was log_2_ transformed. We classified tumors into subgroups most resembling Basal-like, Luminal A, Luminal B, HER2-enriched or Normal-like breast cancer based on PAM50 genes by applying the calling algorithm developed by Parker et al. [17].

### Statistical analysis

Outcomes of the study were defined as recurrence/breast cancer-specific mortality (disease-free survival: DFS) and/or all-cause mortality (overall survival: OS). (Note: recurrence is not collected in NBHS or SCCS, and therefore DFS can only be investigated in the SBCSS.) Event-free participants were censored at the date of last follow-up. The associations between *ALDH1A1* expression levels and DFS and OS were evaluated using a Cox regression model with adjustment for age at diagnosis, TNM stage, chemotherapy treatment, radiotherapy treatment, and basal-like breast cancer subtype in the SBCSS and in other studies whenever available. The Kruskal-Wallis test was used to compare *ALDH1A1* mRNA expression among different tumor grades and stages. The Wilcoxon-Mann-Whitney test was used to analyze *ALDH1A1* mRNA expression in patients with TNBC/non-TNBC and patients with basal-like TNBC and non-basal-like TNBC.

In addition, 4 publicly-available TNBC microarray datasets: GSE25065 (*n* = 64) [23], GSE25055 (*n* = 95) [23], GSE21653 (*n* = 87) [24], and a combined data set (*n* = 101) of GSE10886, GSE6128, GSE3165 and GSE3521 [25-28], were included in the meta-analysis. Original gene expression data were log_2_ transformed. Clinical data and gene expression data were obtained from publicly-available data sets from previous publications and from the Gene Expression Omnibus (GEO) deposited at the National Center for Biotechnology Information (NCBI). To ensure that we derived only high-quality survival data sets from the published breast cancer studies, we applied the “rule of fifty” [29-31] as an inclusion criterion. Specifically, to be included in our study, each dataset was required to have at least 50 TNBC samples with survival data and a minimum of 10 events, as well as 60% or more of its samples with survival information. In total, 10 datasets with molecular subtype information and survival information were identified from the NCBI database. Among them were 3 independent data sets, GSE21653 [24], GSE25055 [23], GSE25065 [23], and 1 combined data set of GSE10886 [25], GSE6128 [26], GSE3165 [27], and GSE3521 [28] that met our study criteria and were thus included in our first-stage screening analysis. Cox regression models were used to derive hazard ratios (HRs) for breast cancer recurrence/breast cancer-specific mortality in association with each mRNA, with adjustment for age at diagnosis and TNM stage. The mRNAs, including ALDH1A1 and ALDH1A3, from the 3 independent data sets and the combined data set, were used as exposure factors in the Cox model, respectively. Each gene was categorized into two categories: < median (reference) and ≥median. Four HRs from each mRNA were used in the meta-analyses. The weighted average HR was calculated using an inverse variance of each HR as the weight. All tests were two-tailed with a significance level of *P* < 0.05. All analyses were performed using SAS statistical software (version 9.3; SAS Institute Inc. NC).
